# Explaining and inducing savant skills: privileged access to lower level, less-processed information

**DOI:** 10.1098/rstb.2008.0290

**Published:** 2009-05-27

**Authors:** Allan Snyder

**Affiliations:** Centre for the Mind, University of SydneyNew South Wales 2006, Australia

**Keywords:** autism, Asperger syndrome, savant skills, exceptional talent, privileged access, transcranial magnetic stimulation

## Abstract

I argue that savant skills are latent in us all. My hypothesis is that savants have privileged access to lower level, less-processed information, before it is packaged into holistic concepts and meaningful labels. Owing to a failure in top-down inhibition, they can tap into information that exists in all of our brains, but is normally beyond conscious awareness. This suggests why savant skills might arise spontaneously in otherwise normal people, and why such skills might be artificially induced by low-frequency repetitive transcranial magnetic stimulation. It also suggests why autistic savants are atypically literal with a tendency to concentrate more on the parts than on the whole and why this offers advantages for particular classes of problem solving, such as those that necessitate breaking cognitive mindsets. A strategy of building from the parts to the whole could form the basis for the so-called autistic genius. Unlike the healthy mind, which has inbuilt expectations of the world (internal order), the autistic mind must simplify the world by adopting strict routines (external order).

## 1. Introduction

My intention here is to propose an explanation for savant skills and to explore the possibility of artificially inducing such skills in healthy, normal individuals. This gives insights into the architecture of the healthy mind, especially why certain abilities are deliberately inhibited from conscious awareness. I suggest that the savant condition occurs as a failure of this top-down inhibition process. To set the stage, I argue in §2 that savant skills are latent in us all.

The savant syndrome is a rare condition in which persons with autistic disorder or other mental disabilities have extraordinary skills that stand in stark contrast to their overall handicap. Savant skills are typically confined to five areas: art, music, calendar calculating, mathematics and mechanical/spatial skills (Treffert 2005). These skills are accompanied by an exceptional ability to recall meaningless detail—memory without understanding (Sacks 2007) and a high incidence of absolute pitch (AP) and synaesthesia.

Snyder & Mitchell (1999) argued that all savant skills, including AP and synaesthesia, reside within everyone, but that they are not normally accessible to conscious awareness. Owing to some atypical brain function, savants have *privileged access* to raw, less-processed information—information in some interim state before it is packaged into holistic labels. This privileged access facilitates a distinct literal cognitive style in which a person thinks in detail, working from the parts to the whole (De Clercq 2003). Savant skills are a form of reproduction. Savants access or read off something that exists in all of our brains, but is normally inaccessible through introspection (Snyder & Mitchell 1999). The precise neuroanatomical mechanism for gaining this privileged access is not yet resolved. It may be associated with an atypical hemispheric imbalance wherein concept networks are bypassed or inhibited.

Accordingly, it was predicted (Snyder, in Carter 1999) and subsequently shown (Snyder *et al*. 2003, 2006; Young *et al*. 2004; Gallate *et al*. 2009) that savant-like skills can sometimes be artificially induced in normal healthy individuals by inhibiting part of the brain—the left anterior temporal lobe (LATL). This is consistent with the notion that autistic savants have some atypical left brain dysfunction or inhibition together with right brain compensation (Miller *et al*. 1998; Treffert 2005; Sacks 2007). In addition, this explanation would appear to fit with contemporary views on hemispheric competition and how disinhibiting the non-dominant hemisphere can artificially offset such competition (Hilgetag *et al*. 2001). It possibly also explains the right-hemispheric bias sometimes associated with autism and other pathologies (§4, below). In other words, everyone has the raw information for savant skills, but it requires a form of cortical disinhibition or atypical hemispheric imbalance to be accessed. As I discuss below, there are various factors that could facilitate this disinhibition.

Apart from attempting to demonstrate that savant skills exist in everyone, there is an additional reason for attempting to artificially induce the savant-like state. Savant skills are normally not creative, being largely imitative. Nonetheless, by being atypically literal, with a tendency to concentrate more on the parts than on the whole, a person sees the world in a less-biased light. Such a cognitive strategy offers advantages for particular classes of problem solving, such as those that necessitate breaking cognitive illusions. A strategy of building from the parts to the whole could form the basis for the so-called autistic genius (Snyder 2004), and provide hints for avenues to artificially enhance creativity as has been discussed elsewhere (Snyder *et al*. 2004).

## 2. Savant skills latent in everyone?

We have argued that savants have privileged access to lower level, less-processed information, before it is packaged into holistic concepts and labels—savants tap into or read off information that exists in all of our brains; but this information is normally beyond conscious awareness owing to top-down inhibition (Snyder & Mitchell 1999; Snyder *et al*. 2004).

This is supported by powerful arguments: those who have protracted experience with savants say that their ‘gift springs so to speak from the ground, unbidden, apparently untrained and at the age of somewhere between 5 and 8 years of age. There is often no family history of the talent’ and it ‘is apparently not improved by practice’ (O'Connor 1989, p. 4). ‘The core ability behind the skill emerges spontaneously and does not improve qualitatively with time even though it might become better articulated’ (O'Connor 1989). In addition, the talents are largely imitative.

If these skills are not latent, it would appear highly coincidental that such a peculiar subset of abilities should be so compelling to a significant fraction of savants across all cultures and also that many of these same savants simultaneously have several savant skills (Rimland & Fein 1988), each of which are similarly peculiar and restricted. Furthermore, autistic savant skills are known to recede or be lost altogether with maturity (Selfe 1977; Barnes & Earnshaw 1995; Treffert 2006).

Savants cannot normally give insights into how they perform their skill and are uncontaminated by learnt algorithms. It just comes to them. They just see it. With maturity, the occasionally offered insights are suspect, possibly being contaminated by the acquisition of concepts concerning the particular skill. Yet, I have labelled one savant, Daniel Tammet, a Rosetta stone (Johnson 2005).

By far, the most compelling argument for savant skills residing equally within everyone is that they can emerge ‘suddenly and spontaneously’ (Miller *et al*. 2000, p. 86) in individuals who had no prior history for them, either in interest, ability or talent (Treffert 2006; Sacks 2007, pp. 157 and 313). Striking examples include skills in art, music (Sacks 2007), mathematics (Treffert 2006, p. 85), calendar calculating (LaFay 1987; Osborne 2003) and possibly AP (Zatorre 1989, see p. 573). The same appears to hold for synaesthesia (Sacks 2007, p. 180), as theory suggested (Snyder & Mitchell 1999), which is reported frequently by autistic savants (Heaton *et al*. 1998; Sacks 2007; Tammet 2007, 2009). Furthermore, these acquired savant skills have been known to diminish with recovery from illness (Sacks 2007, p. 315).

Acquired savants arise from a variety of causes (Treffert 2006) including left frontotemporal dementia (Miller *et al*. 1998), physical injury to the left temporal lobe (LaFay 1987; J. Hirsch & A. Snyder 2005, personal communication), left hemispheric strokes (Sacks 2007, p. 315), severe illness to the central nervous system (Treffert 2006) and even when under the influence of hallucinogens (Humphrey 2002; Sacks 2007, p. 181).

## 3. Inducing savant skills artificially

Taken together, the above facts argue persuasively that savant skills reside within us all and that they can be rapidly switched on and off by natural causes. But, can they be induced temporarily by artificial means?

‘Although we do not normally have access to lower levels of information as do savants, is there nonetheless some artificial means to promote this access’ (Snyder & Mitchell 1999), say, by inhibiting part of the brain with magnetic pulses to inhibit top-down inhibition? (see Snyder's suggestion in Carter 1999).

There are now several accounts of artificially induced savant-like skills, in drawing, proofreading, numerosity and false memory reduction, all by inhibiting the LATL with repetitive transcranial magnetic stimulation (rTMS; Snyder *et al*. 2003, 2006; Young *et al*. 2004; Gallate *et al*. 2009).

Low-frequency rTMS temporarily inhibits neural activity in a localized area of the cerebral cortex, thereby creating ‘virtual lesions’ (Hilgetag *et al*. 1999; Walsh & Cowey 2000; Hoffman & Cavus 2002; Steven & Pascual-Leone 2006). As discussed below in §4, the LATL is implicated in the savant syndrome for both autistic savants as well as savants who emerge late in life as a result of frontotemporal lobe dementia (Miller *et al*. 1998, 2000; Hou *et al*. 2000).

### (a) Induced drawing skills

We cannot draw naturalistic scenes unless we are taught tricks (Gombrich 1960). This is surprising because our brains obviously possess all of the necessary visual information required to draw, but we are apparently unable to consciously access it for the purpose of drawing (Snyder & Thomas 1997; Snyder & Mitchell 1999). Unlike artistic savants (Selfe 1977; Wiltshire 1987; Miller *et al*. 1998), we tend to be more aware of the meaningful whole than its constituent parts.

Snyder *et al*. (2003) directed low-frequency rTMS for 15 min over the LATL of 11, right-handed, healthy participants. The participants were given 1 min to draw a dog, horse or face from memory, before, during, immediately after and 45 min after rTMS treatment.

Magnetic stimulation caused a major change in the schema of the drawings of 4 out of 11 participants. Two of these also underwent sham (inactive) stimulation either the week before or after the real test. The changes in drawing style were observed *only* following active stimulation and not after sham stimulation. In some cases, the drawings returned to ‘normal’ 45 min after rTMS ceased. Young *et al*. (2004) also reported rTMS-enhanced drawing skills.

Several participants reported greater awareness of detail in their surrounds after active rTMS. One participant published his experience, stating that he ‘could hardly recognize the drawings as his own even though he had watched himself render each image’. (Osborne 2003, p. 38).

### (b) Induced proofreading skills

It is easy to miss errors of writing in a familiar passage. Presumably, our propensity to impose meaning inhibits our awareness for the details that comprise the meaning (Bartlett 1932). So, in an attempt to artificially induce autistic-like literalness, Snyder *et al*. (2003) had the above 11 participants undergo a test for proofreading following the same rTMS protocol. Without rTMS, participants almost always missed a duplicated word, such as ‘the’ in familiar proverbs, even after multiple exposures.

Two participants displayed a noticeable improvement in their ability to recognize duplicated words in text following stimulation. They did comparatively well during and/or immediately after stimulation and comparatively poorly both before and 45 min after. Importantly, these two participants also displayed pronounced style changes in their drawings during and after real stimulation but not after placebo stimulation. None of the participants improved at proofreading with placebo stimulation.

In conclusion, low-frequency rTMS of the left frontotemporal lobe caused major changes in the schema of drawings for 4 out of 11 participants, 2 of whom significantly improved at proofreading.

### (c) Induced numerosity

It is not possible to accurately estimate a large number of objects without counting them successively. A small number, three or four, can be accessed (Jevons 1871). Yet, there have been reports over time about the ability of autistic savants to accurately guess large numbers of objects (Scripture 1891; Sacks 1986; Treffert 2006). For instance, Sacks (1986) observed autistic twins who instantly guessed the exact number of match sticks that had just fallen on the floor, saying in unison ‘111’. Such reports motivated Snyder *et al*. (2006) to induce savant-like numerosity abilities in 12 right-handed participants.

**Figure 1:**
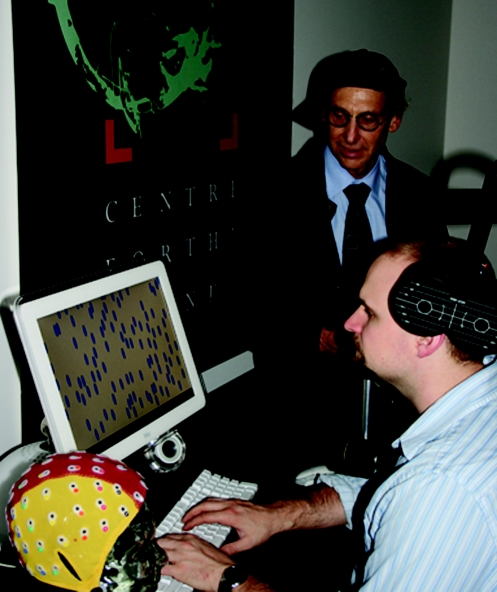
TMS set-up for the numerosity experiment.

Low-frequency rTMS was applied to the LATL for 15 min (Snyder *et al*. 2006). Participants were presented with between 50 and 150 discrete elements on a monitor with rTMS and sham stimulation. Each session involved 60 trials, that is 20 opportunities to guess the number of elements before, immediately after and 1 hour after rTMS. The exposure time was 1.5 s: too short for anyone to count the number of elements, but sufficiently long to resemble exposure times in real-life situations (figure 1).

**Figure 2:**
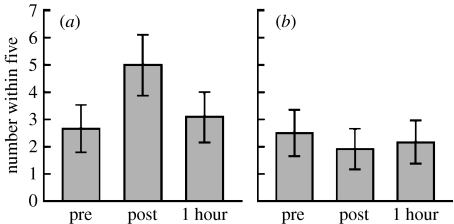
Mean ability across all participants to make guesses within the bulls-eye criterion of 5 with (*a*) TMS and (*b*) sham. Shows numerosity performance before (pre), immediately after (post) and 1 hour after rTMS. Error bars represent 95% confidence intervals and 99% of estimates were multiples of five (from Snyder *et al*. 2004).

Out of 12 participants, 10 improved their ability to accurately guess the number of discrete elements immediately following magnetic pulse stimulation. Out of these 10 participants, 8 became worse 1 hour later, as the effects of the magnetic pulses receded. None of those eight participants exhibited that pattern during the sham session. The probability of as many as 8 out of 12 people doing the best just after rTMS and not just after the sham by chance alone is less than 1 in 1000 (*p*=0.001; figure 2).

#### (i) Why does becoming more literal enhance numerosity?

We argue that the estimation of number by normal people is performed on information after it has been processed into meaningful patterns. The meaning we unconsciously assign to these patterns interferes with our accuracy of estimation, whereas savants, by virtue of being literal, have less interference.

This is consistent with the fact that the accuracy of estimating numbers of elements depends on their arrangement (Ginsburg 1976, 1991; Dehaene 1997)—‘perceived numerosity depends more on higher level cognitive factors… than on lower level perceptual or sensory factors’. (Krueger 1984, p. 540).

This insight has an important generalization. The healthy brain makes hypotheses in order to extract meaning from the sensory input, hypotheses derived from prior experience (Gregory 1970, 2004; Snyder & Barlow 1988; Snyder *et al*. 2004). So judgements in general are likely to be performed on this hypothesized content, not on the actual raw sensory input. This suggests the possibility of artificially reducing certain types of false memories and prejudice by making a person more literal, as well as enhancing creativity (Snyder *et al*. 2004).

### (d) Reducing false memories

It is well known that our memories are not literal representations of the past. Instead, ‘facts’ are unconsciously constructed to fit our schemata (Loftus 2003; Schacter & Addis 2007). Yet, certain pathologies, including autism and anterior temporal lobe (ATL) dementia (Beversdorf *et al*. 2000; Simons *et al*. 2005; Hillier *et al*. 2007), can lead to literal recall and thus greater resistance to false memories.

This inspired Gallate *et al*. (2009) to reduce false memories by temporarily inhibiting the LATL in 14 normal participants, using low-frequency magnetic pulse stimulation. The false memory paradigm of Roediger & McDermott (1995) was adopted, with stimulation applied between the study and test phases of the task.

After stimulation, participants had 36 per cent fewer false memories and intact veridical memory, a result that is comparable with the improvement that people with autism and semantic dementia show over normal individuals.

## 4. The role of the left anterior temporal lobe in the savant syndrome

Why did we apply rTMS to the LATL? The savant syndrome is often associated with some form of left brain dysfunction together with right brain compensation, leading to a predilection for literal, non-symbolic skills (Sacks 2007, pp. 314–315; Treffert 2005, 2006). This is consistent with the role of the left hemisphere in hypothesis formation: the left, but not the right, hemisphere tends to search for patterns, and match them to prior experience (Wolford *et al*. 2000). Furthermore, most savants are autistic and autism has sometimes been associated with a right-hemispheric bias (Herbert *et al*. 2005; Koshino *et al*. 2005) and a left hemisphere dysfunction (Wilson *et al*. 2007).

The LATL has been specifically implicated in the savant syndrome, both for an autistic savant as well as for individuals who become savants at the onset of frontotemporal dementia (FTD; Miller *et al*. 1998, 2000; Hou *et al*. 2000). Patients with FTD displayed autistic savant-like artistic skills where none existed, along with other autistic traits such as preoccupation with visual details and a loss of semantic memory. Miller *et al*. (1998) conclude that, ‘loss of function in the LATL may lead to “paradoxical functional facilitation” of artistic and musical skills’.

Compelling evidence also exists for the ATL as the critical substrate for semantic representation, encompassing the memory and meaning of all types of verbal and non-verbal stimuli—words, pictures, objects and faces (Pobric *et al*. 2007). The LATL is especially vital for semantic processing, implicated as the region responsible for conceptual knowledge, labels and categories (Miller *et al*. 1998; Mummery *et al*. 2000; Thompson *et al*. 2003; Gainotti 2007; Noppeney *et al*. 2007; Olson *et al*. 2007).

When the LATL is damaged, patients lose their semantic memory and their ability to name or label objects, while retaining the ability to recall object details (Mummery *et al*. 2000; Gainotti 2007). Oliveri *et al*. (2004) found that participants were less accurate in interpreting the meaning of opaque idioms (they became more literal) after rTMS to the left temporal lobe. Finally, the core features of semantic dementia have been induced by inhibiting the LATL with rTMS. Inhibition of LATL in normal participants can temporarily lead to semantic impairment in picture and word comprehension tasks, mimicking symptoms of semantic dementia—‘with impairment to the ATL, core semantic representations become degraded and patients are unable to activate all of the information associated with a concept’ (Pobric *et al*. 2007, p. 20 139).

## 5. Why rTMS improves a person's savant-like ability?

It is interesting to speculate on how rTMS, or damage to the LATL, could give rise to savant-like skills. One theory is that, in the normal brain, the conceptual networks (concerned with meaning and labels) tend to inhibit networks concerned with detail (Snyder *et al*. 2004). By inhibiting these networks, we may facilitate conscious access to literal details, leading to savant-like skills.

By obscuring the meaning of something, we become more aware of the details that comprise it. It is easier to draw a face if its meaning is suppressed, for example, by turning the face upside down (Edwards 1989). Inhibiting concept networks could disinhibit networks that are receptive to novel detail, as foreshadowed by Kapur (1996), Miller *et al*. (2000) and Hilgetag *et al*. (2001). Both hemispheres contribute to semantic memory, but the right hemisphere appears to have a greater role in novel meanings (Goldberg 2005; Goel *et al*. 2007; Sacks 2007, p. 155; Pobric *et al*. 2008).

The possibility that cortical areas responsible for concepts could inhibit those concerned with detail is consistent with top-down processing (Summerfield *et al*. 2006; Furl *et al*. 2007) and with evidence about hemispheric competition (Kapur 1996; Miller *et al*. 2000; Sacks 2007, p. 155), as is also the possibility of reversing the inhibition by suppressing the dominant cortical area with rTMS (Oliveri *et al*. 1999, 2004; Hilgetag *et al*. 2001; Theoret *et al*. 2003; Sack *et al*. 2005). In particular, Hilgetag *et al*. (2001) concluded that ‘competition between different brain structures might, thus, be a general principle of brain function (Walsh *et al*. 1998) and may explain the paradoxical behavioural enhancement or recovery observed after various brain lesions’ (Kapur 1996; Hilgetag *et al*. 1999).

## 6. Discussion

I have argued that the extraordinary skills of savants are latent in us all and that they can be induced artificially owing to the inhibiting influence of low-frequency rTMS, that is, by turning off part of the brain, not by exciting it. My hypothesis is that savant skills are facilitated by privileged access to raw, less-processed sensory information, information that exists in all brains but is inaccessible owing to top-down inhibition. Thus, autistic savants tend to see a more literal, less filtered view of the world. Their ‘skill’ or performance does not depend on active learning, but simply on an effortless ‘reading off’ of this less-processed information.

Sensory hypersensitivity and enhanced perception of details (Minshew & Hobson 2008) are a direct consequence of privileged access. AP is another. Although we all have the necessary frequency analysers, AP cannot be learned and yet it is common among savants. The same goes for naturalistic drawing skills, for recall of seemingly meaningless details and for other savant performance (Snyder & Mitchell 1999).

Finally, it should be said that our ‘privileged access’ hypothesis remains to be proven. The empirical evidence so far, while consistent with the hypothesis, is preliminary and requires independent researchers to replicate the findings. In this regard, there are many factors that could frustrate attempts to artificially induce savant skills with low-frequency rTMS (e.g. see Robertson *et al*. 2003). This could in part explain why savant skills are not induced uniformly in everyone. Furthermore, networks in addition to those of the LATL may be implicated.

Why are savant skills suppressed in normal individuals? And, why is it that all autistic individuals are not savants?

### (a) Why are savant skills normally suppressed?

If we all have latent savant skills, why are they not normally accessible? Perhaps they are deliberately inhibited as a principle of economy—object attributes are inhibited from conscious awareness once a label (concept) is formed (Snyder *et al*. 2004). After all, it is the object label or its symbolic identification that is of ultimate importance and not the actual attributes derived by the brain to formulate the label (Snyder & Barlow 1998; Snyder & Mitchell 1999). There is no need to be consciously aware of such details, which explains why we cannot draw natural scenes without being taught the tricks to do so. This strategy accelerates decision-making, especially when confronted with only partial information (Snyder *et al*. 2004). It might also accelerate the process of learning because, without grouping information into meaningful packets, the brain is overwhelmed (Seidenberg *et al*. 2002).

### (b) Why are all autistic people not savants?

The majority of savants are autistic. Why not all? Autistic spectrum disorders encompass a hugely diverse population. However, it may well be that autistic savants represent autism in its purest form, uncontaminated by learned algorithms and other disorders that are frequently associated with autism. In other words, autistic savants typify an idealized, pure autism, most closely identified with Kanner's (1943) infantile autism—a mind in a protracted state of infancy (Snyder *et al*. 2004), a preconceptual mind that thinks in detail, rather than through concepts. This oversimplifying caricature goes some way to explain why all autistic people are not savants.

### (c) Privileged access: a unifying theory of autism?

The label weak coherence (Happé & Frith 2006) and/or lacking theory of mind (Baron-Cohen *et al*. 1985) aptly captures a collection of autistic traits that were first introduced by Kanner (1943)—the *what* of autism—but they do not provide an explanation for the *why* of autism. I suggest that the state of pure (infantile) autism is a failure in the process of concept formation, and its associated top-down inhibition of attributes that comprise concepts, which may offer a mechanism that could unite the present descriptive theories. We have access to models of the world (‘mindsets’ or mental templates) that embody the familiar. These allow us to manoeuvre rapidly when confronted with only partial information. Concepts order the world internally. Without them, order must be imposed externally, hence the imposition of rigid routines that characterizes infantile autism.

### (d) Autistic genius: a consequence of privileged access?

A fundamental bottleneck to creativity is our inability to join the dots up in novel ways. We have a predisposition to impose prior connections (§3*c* above). But, creativity would seem to require that we, at least momentarily, free ourselves of previous interpretations. Such literalness is a consequence of privileged access and thus gives insights into the so-called autistic genius (Snyder 2004) as well as hints to artificially enhance creativity (Snyder *et al*. 2004).

The classical portrait of autism is that of rigid insistence on sameness, rote memory and significant learning disabilities. Even autistic savants are the antithesis of creative, being largely imitative: ‘there are no savant geniuses about… Their mental limitations disallow and preclude an awareness of innovative developments’. (Hermelin 2001, p. 177).

Are there instances when privileged access facilitates the creative process? Asperger (1944/1991) spoke of autistic intelligence as being the intelligence of true creativity, adding that it seems that for success in science or art, a dash of autism is essential. And, according to Fitzgerald (2004), a number of intellectual giants had autistic traits.

The fact that genius might fall within the autistic spectrum challenges our deepest notions of creativity. Are there radically different routes to creativity: normal and autistic? The autistic mind builds from the parts to the whole—a strategy ideally suited to working within a closed system of specified rules. By contrast, the ‘healthy’ mind appears to make unexpected connections between seemingly disparate systems, inventing entire new systems rather than finding novelty within a previously prescribed space (Snyder 2004).
